# Colorism and Sexual Health: A Rapid Review

**DOI:** 10.3390/ijerph23070916

**Published:** 2026-07-17

**Authors:** Gina Diagou Sissoko, Ariana Jaspal, Sofia Walters, Jasmine Abrams

**Affiliations:** 1 Yale School of Public Health, Yale University, New Haven, CT 06510, USA; 2 Department of Psychology, Tufts University, Medford, MA 02155, USA

**Keywords:** colorism, skin tone, sexual health, sexual agency, social determinants of health

## Abstract

**Highlights:**

**Public health relevance—How does this work relate to a public health issue?**

- Colorism (or discrimination based on skin tone) is a pervasive but understudied determinant of sexual health that disproportionately affects Black and Brown girls globally.
- This rapid review synthesizes evidence linking skin-tone bias, discrimination, and internalized colorism to sexual health outcomes, filling a critical gap in the social and structural determinants of the sexual health literature.

**Public health significance—Why is this work of significance to public health?**

- Despite the global prevalence of colorism, we identified only eight studies exploring its connection to sexual health, indicating an evidence gap with significant implications for health equity among marginalized girls and women.
- The existing research highlights consistent mechanisms such as self-esteem, body image, and discrimination-related trauma and stress, showing that colorism shapes sexual health through pathways that are largely unexplored by current public health frameworks.

**Public health implications—What are the key implications or messages for practitioners, policy makers and/or researchers in public health?**

- Sexual health researchers and practitioners should integrate colorism-informed frameworks into their work, using validated skin-tone/colorism measures, globally diverse samples, and outcome frameworks that include positive health outcomes alongside sexual risk.
- Policy makers should recognize that colorism is a structural determinant of sexual health inequity and prioritize adapting and developing colorism-informed sexual health interventions, particularly for those groups that are most vulnerable to harm.

**Abstract:**

Colorism is a skin-tone stratification system that privileges proximity to Eurocentric features and disadvantages darker skin tones and Afrocentric features. Although colorism is increasingly recognized as a gendered social determinant of health, its role in shaping sexual health among Black and Brown girls and women remains understudied. This rapid review synthesized peer-reviewed empirical evidence on the relationship between colorism, skin tone, and sexual health outcomes among girls and women. Searches were conducted in PsycINFO and PubMed between April and May 2026. After removing duplicates, 114 unique records were screened in Covidence, yielding eight eligible studies. Five studies were quantitative, two were qualitative, and one was mixed methods. The included studies were synthesized narratively due to heterogeneity in study design, measurement, and outcomes. Findings suggest that colorism is associated with sexual risk behaviors, sexual agency and desirability, sexual development, and HIV-related sexual functioning. Across studies, colorism appeared to shape sexual health through three interrelated pathways: internalization and self-esteem, colorist desirability hierarchies that influence sexual agency and relationship dynamics, and differential sexualization experiences that shape girls’ sexual development and vulnerability across the life course. One study also highlighted parental support as a potential protective factor. Collectively, the findings position colorism as a potentially important social determinant of sexual health and underscore the need to integrate colorism into future sexual health research, prevention, and intervention frameworks.

## 1. Introduction

Sexual health disparities remain a significant public health concern among Black and Brown women and girls, who experience disproportionate rates of HIV, sexually transmitted infections, sexual victimization, and other adverse sexual health outcomes relative to their White counterparts [1,2,3]. Although research has increasingly examined the role of structural and social determinants in shaping these inequities, one factor that has received comparatively little attention is colorism. Colorism is a skin-tone stratification system that privileges lighter-skinned people of color in closer proximity to Eurocentric physical features and disadvantages darker-skinned people with more Afrocentric features [4,5]. It particularly impacts Black and Brown women, as the phenomenon is rooted in colonial and slavery-era practices that conferred preferential opportunity on lighter-skinned individuals (e.g., household vs. field labor), solidifying intra-racial hierarchy across generations [4,6,7]. In the United States, darker skin tone has been associated with lower income, education, and occupational status [8,9]; harsher criminal sentencing [10]; higher racial discrimination [11,12]; and higher mortality [13]. Similar to racism, colorism acts as a significant but understudied social determinant of health [5,9], where individuals with darker complexions experience worse outcomes than those with lighter complexions [14].

Colorism is a gendered phenomenon, as it impacts men and women differently [7,15,16]. Light skin tone operates as a form of social capital for women. Lighter-skinned women earn higher salaries, attain higher levels of education, are perceived as more attractive, and are more likely to marry partners of higher socioeconomic status [7,17,18]. Darker-skinned women and girls, on the other hand, are perceived as less attractive and are more likely to be bullied, socially excluded, and criminalized [18,19,20,21]. Among young Black women specifically, darker skin tone has been associated with lower educational attainment, lower maternal education, reduced social support, lower income, and reliance on government health insurance [16]. The health consequences of colorism are also evident, as dark-skinned Black women experience higher cumulative biological risk and poorer self-rated health [22].

Colorism also has implications for mental health. In-group colorism has also been associated with a greater risk of obtaining diagnoses of lifetime psychiatric disorders, alcohol and substance use disorders, anxiety disorders, eating disorders, and suicidal ideation [23,24]. Among Black women and girls specifically, colorism has been linked to suicidal ideation [25], substance use [26], binge eating [27], skin-tone dissatisfaction and skin bleaching behaviors [28,29,30], and lower self-esteem [5,31,32]. Darker-skinned women are at higher risk for these outcomes, given that they experience the highest levels of both in-group and out-group skin tone colorism [11]. Although the mental health consequences of colorism are increasingly documented, far less is known about how colorism shapes sexual health outcomes.

### Colorism and Sexual Health

Black and Brown girls and women experience a heightened burden of sexual health risks. They experience disproportionate rates of negative sexual health outcomes, such as STIs/HIV, teen pregnancy, and earlier sexual debut, as well as greater sexual victimization in comparison with their White counterparts [1,2,3]. There are several theoretical pathways that may link colorism to sexual health outcomes. First, objectification theory describes how habitual body monitoring and the internalization of beauty ideals can lead to body shame, which may reduce sexual agency [33]. In the context of colorism, beauty ideals include Eurocentric standards of beauty (e.g., preference for lighter skin tone) [28], which may lead to risky health behaviors and outcomes. Second, skin tone trauma theory suggests that colorist incidents accumulate and produce trauma-related symptoms and stress, which in turn lead to adverse health behaviors and outcomes [5]. Furthermore, theories around the adultification of Black girls, where they are perceived as older, more knowing, and less innocent than White peers, have implications for early sexualization [34]. Finally, colorism intersects with racist social tropes and stereotypes about Black and Brown women, which characterize women and girls as innately hypersexual or docile based on skin tone, potentially impacting their sexual socialization [19,26,35].

Despite prior reviews on colorism and mental health [36,37], to our knowledge, there has been no synthesis on the relationships between colorism and sexual health specifically. Because colorism operates as a stratification system within global communities of color and not just between groups, it is distinct from racism. As such, colorism has unique implications for sexual health risk prevention and intervention research [5,38]. The aim of this rapid review was to synthesize peer-reviewed empirical evidence on the relationship between colorism, skin tone, and sexual health behaviors and outcomes.

## 2. Method

This study employed a rapid review methodology to synthesize existing evidence on the relationship between colorism and sexual health outcomes among girls and women. A rapid review approach was selected to allow for a structured yet time-efficient synthesis of an emerging and relatively understudied body of the literature. Rapid reviews are evidence-synthesis research designs that use systematic methods to identify, appraise, and synthesize the existing literature within a shortened timeframe [39,40]. The review incorporated both quantitative and qualitative studies and used a narrative synthesis approach due to expected heterogeneity in study designs, measures, and outcomes.

### 2.1. Search Strategy

A structured literature search was conducted in PsycINFO and PubMed. The search strategy was developed to capture three key domains: (1) colorism and skin tone, (2) sexual health and sexual risk outcomes, and (3) female populations. The following search string was used:

1. (colorism OR shadeism OR “skin tone” OR “skin color” OR complexion OR “skin tone discrimination”);
2. AND (“sexual risk” OR “sexual behavior” OR “condom use” OR “unprotected sex” OR HIV OR STI OR “sexual debut” OR “number of partners”);
3. AND (girl* OR women OR female*).

**Figure 1:**
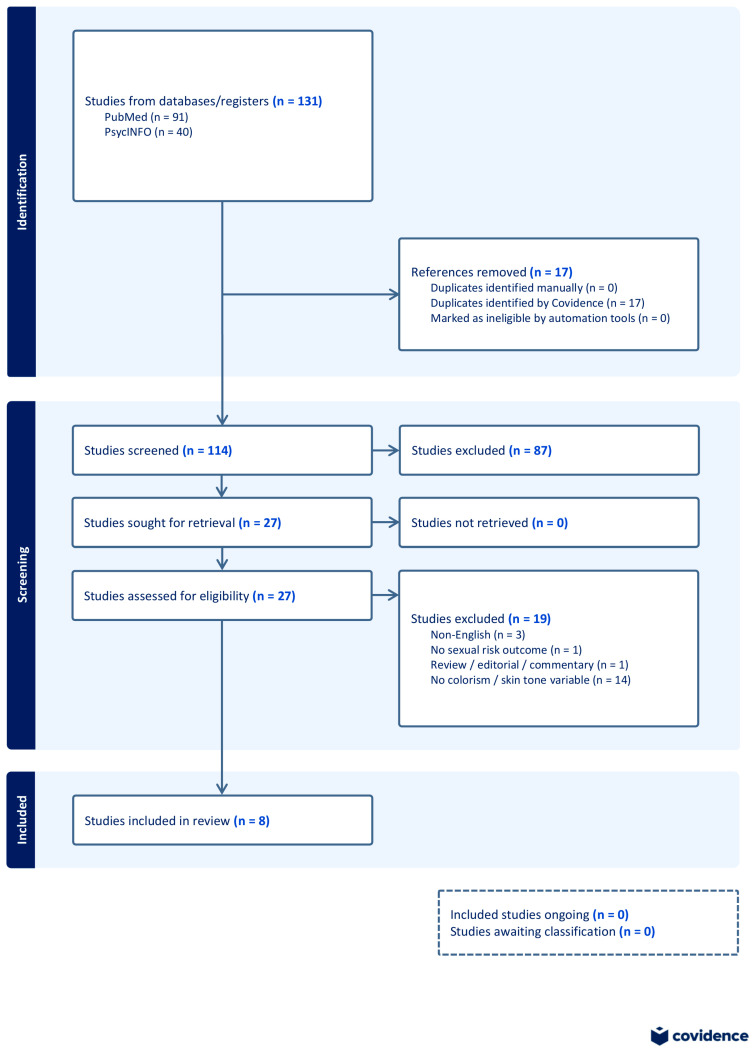
Preferred Reporting Items for Systematic Reviews and Meta-Analyses (Prisma) Flow Diagram.

The final search (May and April 2026) yielded 91 records from PubMed and 40 records from PsycINFO (see Figure 1). After the removal of 17 duplicate records, a total of 114 unique records were retained for screening. All records were imported into Covidence for screening and data management.

### 2.2. Eligibility Criteria

Studies were included if they (1) were peer-reviewed empirical studies (quantitative, qualitative, or mixed-methods), (2) examined colorism, skin tone, or related constructs (e.g., complexion, shadeism) and (3) included at least one sexual health or sexual risk outcome (e.g., sexual behavior, STI/HIV outcomes, sexual development, sexual attitudes, or sexual victimization). Studies also had to include girls and/or women in the sample and needed to be published in English. Given the limited literature in this area, the age range was not restricted. Studies were excluded if they did not include a measure or discussion of colorism or skin tone, did not include any sexual health-related outcome, were reviews, editorials, commentaries, or theoretical papers, included only male participants, or were not published in English.

### 2.3. Study Selection

Title and abstract screening were conducted by two reviewers using Covidence. Studies that met the inclusion criteria or were unclear based on the abstract were advanced to full-text review. Given the small and emerging nature of the literature, screening decisions were intentionally inclusive. Full-text screening and quality assessment were conducted to confirm eligibility. Discrepancies or uncertainties were resolved through consensus discussion involving three reviewers.

### 2.4. Data Extraction

Data extraction was conducted using a standardized extraction template developed in Covidence. Extracted information included: study characteristics (e.g., study type, design, country), sample characteristics (e.g., sample size, age, population), measures or descriptions of colorism, sexual health outcomes, main findings linking colorism to sexual health, direction of associations for quantitative studies, key themes for qualitative studies, study-level interpretation of findings, and noted limitations. Each study was extracted by a single reviewer, and all extractions were subsequently reviewed and verified by a second reviewer to ensure accuracy and consistency.

### 2.5. Data Synthesis

Given the heterogeneity in study designs, measures, and outcomes, a narrative synthesis approach was used. Quantitative findings were summarized by the direction and significance of associations between colorism and sexual health outcomes. Qualitative findings were synthesized thematically to identify recurring patterns related to how colorism shapes sexual experiences, perceptions, and behaviors. Findings from quantitative and qualitative studies were then integrated to provide a comprehensive understanding of how colorism relates to sexual health among girls and women.

## 3. Results

### 3.1. Overview

In our rapid review, eight studies examined links between colorism and sexual health outcomes (Table 1). Of these studies, five were quantitative, two were qualitative, and one was a mixed-method study. The total number of participants across studies was *n* = 5070. Seven studies were conducted in the United States and one in Brazil. Three studies focused on adolescent girls, three on adult women, one was a longitudinal study following girls into young adulthood, and one was a mixed-gender study. The sexual health outcomes of the studies spanned sexual risk behaviors, sexual development, sexual agency and desirability, sexual attitudes, and HIV-related sexual functioning.

### 3.2. Colorism Measurement

Across the included studies, colorism was conceptualized and measured in three different ways (Table 1). Three studies used qualitative discussions, exploring participants’ experiences with and exposure to messaging about colorism and skin tone biases and preferences. One study used the Image Acceptance Measure (IAM; [41]) to assess participants’ acceptance of stereotypically preferred physical traits rooted in standards of beauty consistent with “colorism” (e.g., light complexion, straight, long hair, and thinness) and their rejection of “traditional” African American standards of beauty. Another study used the Colorist Attraction subscale (measuring romantic preference for lighter skin tones) and the Skin Tone Self-Concept subscale (measuring salience of skin tone to self-concept) of the In-Group Colorism Scale [42].

Five of the included studies specifically assessed participants’ skin tones. Three of these used self-reported skin tone scales, and the other two used interviewer-rated assessment measurements. In one interviewer-rated skin tone study, skin tone was assessed on a five-point scale (1 = black, 2 = dark brown, 3 = medium brown, 4 = light brown, and 5 = white), while in the other, trained raters coded skin tone on a 0–5 scale (0 = very light, 5 = very dark). In the studies using self-report measures, skin tone was either assessed categorically (Indigenous, Black, Brown, or White), on the ten-point New Immigrant Survey scale (using images of hands ranging from very light to very dark), or on a three-point scale (light skin, dark skin, neither light nor dark skinned).

### 3.3. Skin Tone and Sexual Risk Behaviors

In this rapid review, three studies looked at skin tone and sexual risk behaviors. In their longitudinal study of 397 African American young women and girls (mean age 10.5–21.5) from the Family and Community Health Study (FACHS), Landor and colleagues [43] showed that darker skin tone was indirectly associated with more risky sexual behavior and negative sexual health outcomes through self-esteem. In this study, skin tone was coded from videotapes by trained raters on a six-point scale. Risky sexual behavior was measured through an aggregate score of four items (ever had sex, age at first sex, sex with a potentially HIV-infected partner, and number of partners). Negative sexual health outcomes were measured through an index score that included pregnancy, abortion, and STI diagnosis. The results indicated that darker skin tone predicted lower self-esteem, which in turn predicted more risky sexual behavior, which consequently predicted more negative sexual health outcomes. Notably, there was no direct path from skin tone to sexual behavior or outcomes, and self-esteem fully accounted for this relationship. The authors also tested the moderating effects of parental support and racial identity. Interestingly, parental support moderated the link between skin tone and self-esteem, such that the negative association between darker skin tone and lower self-esteem was weaker among women with higher parental support, indicating that parental support may be a protective factor. Racial identity, however, had no effect on the relationship. Additionally, while the authors recruited from economically diverse samples and controlled for family structure (e.g., single-parent household vs. other family types), socio-economic status was not included in the analysis.

**Table 1:** Summary of included studies.

Authors	N	Age	Country	Design	Colorism Measure	Sexual Health Measure	Sexual Health Outcome
Dantas et al. [44]	387	*M* = 50.85 ^1^	Brazil	Cross-sectional	Self-reported skin color	Sociodemographic, Epidemiological and Clinical Form for PLHIV; Female Sexual Function Index (FSFI, [45])	Dysfunction and Hypoactive Sexual Desire
Teran et al. [46]	539	Range: 18–29	USA	Cross-sectional	Self-reported Skin Tone (In-Group Colorism Scale)	Adapted version of the Sexual Risk Survey [47]	Sexual Risk-Taking Behavior
Bond et al. [48]	26	Median: 20 Range: 18–25	USA	Mixed methods	Qualitative Discussion	-	Sexual Agency and Desirability
Townsend et al. [35]	270	M = 13 Range: 10–15	USA	Cross-sectional	Image Acceptance Measure	JSI Women’s Form; JSI Youth Form—Condom Efficacy subscale; JSI Youth Form—Sexual Risk Behaviors scale	Sexual Attitudes, Condom Efficacy, Sexual Intent
Landor & Halpern [49]	3406 women	Wave 3: M = 21.1 Wave 4: M = 28.2	USA	Longitudinal	Interviewer-rated skin tone (5-pt)	-	Number of past sexual partners and concurrent partners
Landor et al. [43]	397	Wave 1: M = 10.5 Wave 5: M = 21.5	USA	Longitudinal	Interviewer-rated skin tone (0–5)	Study-constructed sexual behavior index; Study-constructed negative sexual health outcomes index (no named validated scale)	Sexual Behavior, STI, Pregnancy, Abortion
Crooks et al. [50]	20	M = 14	USA	Qualitative	Qualitative Discussion	-	Perceptions of Sexual Development
Crooks et al. [51]	25	Range: 9–18	USA	Qualitative	Qualitative Discussion	-	Perceptions of Sexual Development

Note. ^1^ age reported for sexually inactive women only.

Next, in their longitudinal study of 6872 never-married heterosexual young adults (3406 women) from the National Longitudinal Study of Adolescent to Adult Health (Add Health), Landor and Halpern [49] found that skin tone moderated the relationship between marriage attitudes and risky sexual behavior among racial/ethnic minority young adults. Skin tone was measured on a 5-point scale, and risky sexual behavior was operationalized as the number of sexual partners and concurrent partners in the past 12 months. Overall, positive marriage attitudes had a stronger protective effect on risky sexual behavior for lighter-skinned compared to darker-skinned individuals. Among women specifically, lighter-skinned African American women had reduced odds of concurrent partnering when they viewed marriage as important, while this association did not hold for darker-skinned African American women. Skin tone also directly predicted sexual behavior among Asian women, though in an unexpected direction, where darker-skinned Asian women reported fewer partners and lower odds of concurrency than their lighter-skinned peers. According to the authors, this pattern may reflect darker-skinned women’s internalization that marriage is less attainable; therefore, even positive marriage attitudes may not impact sexual behavior in a significant way.

Lastly, one study connected skin tone to hypoactive sexual desire, described as the absence of or marked reduction in desire or motivation to engage in sexual activity [44]. In their cross-sectional study of 387 women living with HIV in Brazil, Dantas and colleagues [44] showed that among sexually active women, self-reported Brown skin color (compared to White, Black, and Indigenous) was significantly associated with hypoactive sexual desire. However, it is important to interpret these findings with caution, as 94.12 percent of participants had hypoactive sexual desire. Furthermore, it is important to note that colorism operates differently across geographical and historical contexts [52], suggesting that skin-color categorizations may also reflect racial categorizations in this study.

### 3.4. Internalized Colorism, Body Shame, and Sexual Risk

This review identified two studies that looked at internalized colorism and sexual risk. Teran et al. [46] conducted a cross-sectional study of 539 self-identified Latinx young adults across the United States to examine the influence of skin tone ideologies on body shame and sexual risk behaviors, with the potentially moderating role of self-esteem. Internalized racism was assessed through two subscales of the In-Group Colorism Scale: the Colorist Attraction subscale, which measures romantic preference for lighter skin tones, and the Skin Tone Self-Concept subscale, which measures salience of skin tone to one’s self-concept. Sexual risk-taking was measured using the adapted Sexual Risk Survey [47]. Results of this study indicated that higher levels of colorist attraction, that is, a preference for lighter skin in romantic partners, were associated with increased engagement with risky sexual behaviors. In addition, colorist attraction was also associated with body shame, suggesting that Latinx young adults who prefer lighter skin tones also feel more negatively about their own bodies. Furthermore, individuals who reported that skin tone was a central part of their self-concept reported more body shame but did not engage in higher levels of sexual risk behaviors. Importantly, self-esteem significantly moderated the relationship between skin tone self-concept and body shame, such that Latinx young adults with average or high self-esteem who placed greater importance on their skin tone reported more body shame. However, this association was not significant among those with low self-esteem.

The second study identified in this review was a cross-sectional study of 270 adolescent girls between the ages of 10 and 15 exploring the interaction of colorism and stereotypic images with identity components in shaping Black girls’ sexual attitudes. Colorism was self-reported using the Image Acceptance Measure (IAM; [41]), which measures endorsement of stereotypically colorist beauty standards (e.g., preference for light skin tone, straight, long hair). Sexual attitudes were measured using a 5-item adapted version of the JSI Women’s Form, assessing the degree to which girls perceived certain sexual behaviors (e.g., unprotected oral sex) as harmful or risky, as well as condom efficacy (use, communication, refusal) and sexual intent (how likely they would be to engage in sexual intercourse in the next three months). The results showed that among Black adolescent girls with a strong sense of ethnic belonging, internalized colorism- that is, believing that lighter skin tone is more beautiful and desirable- was associated with greater intent to have sex in the next three months.

### 3.5. Colorism, Desirability, and Sexual Agency

In our review, one study examined the role of colorism in perceptions of desirability and sexual agency. In their qualitative study of 25 Black women between the ages of 18 and 25, Bond et al. [48] explored the relationship between colorism, media portrayals, and racialized sexual stereotypes as they impact sexual health and behaviors among Black women. Women reported that societal power structures and media portrayals perpetuate colorist hierarchies of desirability, where Eurocentric beauty standards (e.g., lighter skin tones, looser curls) impact how Black women are perceived to be desired in romantic and sexual encounters. For darker-skinned women in particular, these experiences impact self-esteem, and they reported feeling less valued, desired, and sexually empowered to assert agency in sexual relationships and healthcare settings. Darker-skinned women consistently reported internalizing scripts indicating that men prefer lighter-skinned women and women with “good hair” (loosely textured curls or straight hair), which impacted not only how they viewed themselves but also how they interacted with potential partners. In addition, in order to achieve sexual and romantic desirability, participants reported that women who do not meet Eurocentric standards of beauty use skin bleaching products and experience pressure to work “extra hard” in order to be seen as desirable. Despite the clear impact of colorism on Black women’s perceptions of desirability, sexual agency, and empowerment, women also reported a shift in reclaiming representation. They reported a meaningful shift in media representation celebrating natural hair and dark skin tones, allowing women to feel “proud,” “powerful,” and “magical,” which in turn helped them challenge negative self-narratives and reclaim sexual agency.

### 3.6. Colorism and Sexual Development

Two studies in this review examined the impact of colorism on Black girls’ sexual development. Crooks et al. [50] conducted a qualitative study of 20 Black girls (aged 11–18) to describe their perspectives on their sexual development process, with particular attention to the influence of stereotype messaging (e.g., colorism) on sexual behaviors and potential HIV/STI risk. All participants described receiving pervasive messages from the media, culture, and families that encouraged a preference for light skin tone and shame regarding darker skin tones. Participants reported that lighter-skinned girls are often labeled as pretty, exotic, and desirable, leading to early sexualization through experiences of being pursued by boys and older men. Darker-skinned girls, on the other hand, report being bullied, teased, and viewed as unattractive and “abrasive,” leading to significant psychological distress as well as early sexual initiation to seek attention, love, and affection they believe is denied to them due to being viewed as unattractive. Participants also believed that historical, societal, and cultural messages are carried through Black girls’ development and may have long-lasting effects on their sexual development. As such, messaging related to lighter skin tone may increase girls’ risk of being sexualized as children, while the consistent bullying of darker-skinned girls may lead to feelings of rejection and shame, which may lead to engaging in increased or premature sexual activity.

Crooks et al.’s [50] findings are consistent with those of Crooks et al. [51], in which researchers conducted a qualitative study of 25 Black girls (aged 9–18) to specifically examine how colorism influences Black girls’ psychological and sexual development. The results indicated that colorism influences Black girls’ sexual development through three interrelated domains: colorist messaging, colorist assumptions and stereotypes, and consequences of colorism. In particular, girls reported that they receive colorist messages from historical (slavery and colonialism), interpersonal (family, peers, community), and environmental (social media, institutions) sources. These messages extended beyond skin tone to include other Afrocentric stereotypes, including facial features and hair texture. In addition, Black girls were exposed to a variety of assumptions and stereotypes based on skin tone, where lighter-skinned girls were often perceived as desirable, acceptable, kind, and as having “pretty privilege” due to racial ambiguity or proximity to whiteness. Dark-skinned girls reported being stereotyped as aggressive, hostile, loud, undesirable, and strong. The strong attribute, in particular, was seen as a liability that reduced the nurturance and protection they received. According to the findings, the consequences of colorism—through the messaging of the colorist assumptions and stereotypes—lead to consequences related to violence and victimization, internalization, and increased sexual risk. In particular, darker-skinned girls were perceived as being at higher risk for bullying and physical violence. They were also viewed as more overdisciplined and over criminalized by institutions while receiving less protection from adults. The second consequence was internalization, where constant exposure to stereotypic messaging leads to lower self-esteem, feelings of helplessness, and harmful behaviors such as skin bleaching to increase desirability and acceptability. Lastly, girls reported a perception that darker-skinned girls face a higher risk of oversexualization and sexual assault due to a perceived lack of protection and societal devaluation. In addition, internalized colorism and resulting low self-esteem may also lead to engagement in risky sexual behaviors to gain male attention or a sense of self-worth. Lighter-skinned girls, on the other hand, face increased sexual risk primarily from fetishization and exoticization of their lighter skin tone. Overall, this study indicated different pathways in which colorism impacts sexual development and risk based on skin tone.

## 4. Discussion

This rapid review aimed to examine the role of colorism in sexual behaviors and health among women and girls. Overall, eight studies were identified. Although limited, this review provided evidence to support the importance of examining colorism as a meaningful determinant of sexual health among women and girls. The results suggest three pathways through which colorism may influence sexual behaviors and risk outcomes: (1) internalization and self-esteem, (2) colorist desirability hierarchies that shape sexual agency and relationship dynamics, and (3) differential sexualization experiences based on skin tone that influence girls’ sexual development and risk across the life course.

Both quantitative and qualitative studies indicated that internalization and self-esteem may represent important mechanisms linking colorism and sexual health outcomes [43,48,50,51]. Landor et al. [43] provided the strongest empirical evidence for this pathway, demonstrating that darker skin tone predicted lower self-esteem, which in turn predicted higher engagement in risky sexual behaviors, and consequently poorer sexual health outcomes. Importantly, self-esteem fully mediated the relationship between skin tone and risky sexual behaviors. Qualitatively, this was supported by reports from women and girls across three qualitative studies. Participants described internalizing societal messages that devalue darker skin tones and position women and girls with darker skin tones as less attractive, desirable, or worthy of protection. Women and girls reported that these experiences negatively impacted their self-worth, self-esteem, and perceptions of desirability, positioning internalization and self-esteem as potential psychosocial pathways through which colorism may influence sexual decision-making and health outcomes [48,50,51].

A second pathway was related to colorist hierarchies of desirability, which influence sexual agency and relationship dynamics. Across the qualitative studies identified in this review, participants described a hierarchy of attractiveness that privileges lighter skin tones and Eurocentric features while devaluing darker skin tones and Afrocentric features [48,50,51]. Participants reported that these hierarchies shaped how women and girls perceived their own desirability and how they believed to be perceived by potential romantic and sexual partners. In addition, Teran et al. [46] found that greater endorsement of colorist attraction, defined as a preference for lighter-skinned romantic partners, was associated with risky sexual behaviors, further suggesting that internalized colorist beauty ideals may shape romantic and sexual decision-making.

Importantly, in Bond et al. [48], darker-skinned women reported that feeling less desired led them to feel less empowered to assert agency in sexual relationships and healthcare settings, indicating that colorism not only has implications for sexual encounters but also for health-seeking behaviors. Furthermore, girls in Crooks et al. [50,51] papers reported that colorist messages influenced perceptions of attractiveness as well as romantic and sexual worth, further supporting that colorism has the potential to shape sexual health through its influence on desirability perceptions and resulting sexual agency. These findings may help contextualize Landor and Halpern’s [49] finding that positive attitudes towards marriage were associated with reduced sexual risk among lighter-skinned but not darker-skinned women, as the authors suggested that darker-skinned women internalize societal messages about romantic desirability and the attainability of marriage.

Third, this review identified unique pathways through which colorism shapes girls’ sexual development through differential socialization and sexual vulnerabilities based on skin tone and ethnic identity. Across studies, participants described exposure to pervasive colorist messaging from families, peers, media, and institutions that communicate who is attractive, desirable, and worthy [50,51]. These messages appeared to influence girls’ sexual attitudes, expectations, and developmental experiences. For example, Townsend et al. [35] found that among Black adolescent girls with a strong sense of ethnic belonging, endorsement of colorist beauty ideals was associated with greater intent to engage in sexual intercourse. Furthermore, Crooks et al.’s [50,51] findings suggested that colorism may place girls on distinct developmental trajectories. Lighter-skinned girls were described as attractive, exotic, and desirable, increasing their vulnerability to early sexualization and fetishization. In contrast, darker-skinned girls were perceived as aggressive, undesirable, and less worthy of protection, making them more vulnerable to bullying, over-discipline, over-sexualization, and sexual victimization. As such, colorism may shape sexual development by exposing youth to different social messages, expectations, and ultimately differential vulnerabilities across the life course.

Despite the identified intersections and links between skin tone, sexual risk, and socioeconomic status [7,22], associations between economic power and sexual risk behavior were not explored or reported in most of the reviewed literature. Townsend et al. [35] controlled for the effects of mother’s and father’s employment as study participants were sampled from similar low-income communities. Teran et al. [46] controlled for socioeconomic class in analyses involving body shame, as it was negatively correlated with having spent more of one’s life in a wealthier socioeconomic class. Dantas et al. [44] note that their observed variance in sexual dysfunction prevalence in WLHIV may be related to socioeconomic differences in different countries, citing that those in better economic situations report greater sexual pleasure and that socioeconomic aspects can be related to sexual autonomy. Notably absent is a direct examination of the bidirectional relationship between skin tone and economic power itself. The documented association between lighter skin tone and higher socioeconomic attainment [7,53] suggests that colorism may shape sexual risk behavior not only through psychosocial pathways such as body shame or self-esteem but also indirectly through its influence on income, economic independence, and, by extension, sexual autonomy and negotiating power within relationships. None of the studies included in this review explicitly explored or tested this pathway, pointing to a significant gap given that economic power is a well-established determinant of women’s capacity to negotiate safer sex practices and resist coercion.

Our findings are consistent with several theoretical frameworks that inform our understanding of the mechanisms by which colorism shapes Black and Brown women and girls’ sexual health. First, objectification theory suggests that the acculturation of girls and women to internalize an observer’s perspective increases opportunities for shame and anxiety and subsequently health risks [33]. This aligns with Townsend et al.‘s [35] finding that, among those with high levels of ethnic identity, the internalization of colorism and colorist messaging impacts the intention to engage sooner in sexual intercourse. Here, internalization of external perspectives impacts sexual attitudes and decision-making in Black girls. This is particularly important in the context of Landor et al. [43] finding that parental support attenuated the negative associations between darker skin tone and self-esteem, which suggests that family relationships may serve as a protective factor against the internalization of colorist messages and their subsequent impact on sexual health outcomes. As such, sexual health interventions geared to Black girls and women must explicitly address the internalization of colorist messaging as a driver or determinant of sexual risk, while also strengthening protective factors, such as parental support, which may buffer against the harmful effects of colorist messaging. Acknowledging that sexual decision-making is intertwined with appearance-based socialization and stress may be critical for reducing sexual health disparities among women and girls.

Our findings are also consistent with Landor & Smith’s [5] skin tone trauma theory, which proposes that colorist incidents elicit traumatic stress reactions, resulting in negative effects on health and interpersonal relationships. Landor et al.’s [43] findings showing that skin tone, through self-esteem, impacts sexual health outcomes support this theory. For example, darker-skinned girls may be at higher risk of exposure to colorist incidents, which in turn impact their self-esteem, leading to higher engagement in risky sexual behaviors and increased negative sexual health outcomes. Our findings underscore a need for trauma-informed approaches that recognize colorism and skin-tone discrimination as a psychological stressor distinct from general racial discrimination, with implications for sexual health research and interventions.

Lastly, our findings expand sociocultural theories exploring the influence of adultification and stereotypical messaging on sexual health and development in Black girls and women [54]. Crooks et al. [50] showed that sociocultural colorist messages are carried through Black girls’ lives and have the potential for long-lasting effects on their sexual development. Crooks et al. [51] highlighted that colorist messaging and stereotypical assumptions increase Black girls’ exposure to violence and sexual risk and behaviors through different pathways based on skin tone. These findings suggest that colorism is deeply embedded in girls’ developmental trajectories by shaping sexual risk and vulnerability.

### 4.1. Limitations

There are several limitations to our review. Despite a broad search strategy, we only utilized two databases, yielding eight studies that met the inclusion criteria. Although colorism is generally understudied [6], the exclusion of dissertations and grey literature may have further narrowed our evidence base. However, the consistency of our findings across the included studies suggests that colorism is an important and understudied factor in sexual health research. The restriction to English-language publications may have excluded relevant global work, particularly in countries with strong colorism bias, where research may be published in other languages. Geographically, the study corpus was US-centric, with no studies conducted in African, Afro-European, Afro-Caribbean, or Asian contexts, and with only one Brazilian sample. This limits the generalizability of our findings, given the diverse and broad scope and operation of colorism across global contexts. Methodologically, most studies relied heavily on self-reported or interviewer-rated skin tone, which has psychometric limitations that have been previously criticized in the colorism measurement literature [52]. Importantly, despite a broad search strategy, sexual health was narrowly assessed across the included studies, with most outcomes focused on risk behaviors and HIV/STI proxies. The absence of research on sexual pleasure, satisfaction, and effectiveness leaves important sexual health dimensions unexplored. Although the small evidence base limits our ability to draw strong causal inferences, the findings provide strong support for the continued investigation of colorism as a social determinant of sexual health and a potential target for intervention.

### 4.2. Implication and Future Directions

Our rapid review establishes colorism as an important social determinant of sexual health among girls and women, yet the evidence base remains underdeveloped relative to its scope and global prevalence. It is significant that our systematic search resulted in only eight eligible studies, indicating the urgent need for scholarly attention to how skin-tone bias and discrimination shape the sexual health trajectories of girls and women around the world. We outline several key recommendations to advance multidimensional scholarship in this field and to encourage the use of quantitative, qualitative, and mixed methods approaches to address these research gaps.

First, there is a need to develop psychometrically validated colorism and skin tone discrimination measures specific to sexual health contexts to guide future quantitative research. Current studies rely heavily on single-item or interviewer-rated skin tone measurements, which limit comparability across studies. Future measurement frameworks should expand to include hair texture as a colorism marker, since texture-based discrimination often operates alongside skin tone bias. Researchers should draw on emerging tools of sexual risk for Black girls, such as the Risk, Adultification, Messaging, and Protection Scale (RAMPS) [54], to look at associations between skin tone, internalized colorism, and sexual risk. This is particularly relevant to Black girls, as colorism has been identified as a determinant of early sexual socialization and sexual development outcomes in this population [50,54].

Second, colorism should be addressed through global frameworks and collaboration. The concentration of US-based studies in the present review represents a critical gap in sample diversity. Colorism is a global phenomenon [4,52], and international research on colorism must extend across regions, including Africa, the Caribbean, Europe, and Asia, using mixed methods approaches to compare and contrast how it operates in ways shaped by historical, structural, and cultural contexts. International and cross-diaspora comparative work has the potential to identify universal as well as distinct colorism intervention targets to augment the overall cultural sensitivity and efficacy of sexual health interventions.

Third, the current evidence base is largely cisheteronormative, indicating the need to include sexual and gender minority girls and women in future research. The compounded and intersecting experiences of colorism and sexual- and gender-minority stigma may make affected individuals particularly vulnerable to negative sexual health outcomes. Future qualitative studies should be intentional in their focus and inclusion strategies to ensure that the growing literature honors lived experiences and does not recreate the exclusions it seeks to address. Furthermore, the field also needs to move away from its near-exclusive focus on risk and negative outcomes and instead consider moving towards strength- and pleasure-based factors to foster positive sexual health. This might include expanding the paradigms focused on sexual agency, pleasure, satisfaction, and assertiveness in the context of colorism.

Fourth, future research on colorism has the potential to test theory-grounded mechanisms, including self-esteem, hair esteem, body shame, and sexualization, to extend existing conceptual frameworks. In order to fully understand how colorism impacts sexual behaviors and outcomes, longitudinal designs are essential. For example, daily diary and Ecological Momentary Assessment (EMA) methodologies should be used to capture the real-time cumulative effects of colorist discrimination on sexual decision-making.

Fifth, future research should examine whether skin-tone-based economic disparities mediate the relationship between colorism and sexual risk behavior, rather than only treating socioeconomic status as a confounding variable to be controlled for.

Finally, one of the most pressing gaps identified through this rapid review is the absence of colorism-informed sexual health interventions. There are no existing frameworks/programs addressing how colorism and skin-tone biases shape the sexual health behaviors, attitudes, and/or well-being of girls and women. Existing sexual health interventions designed for adolescent girls (e.g., IMARA, IMAGE, SiHLE) should be adapted to incorporate colorism-relevant aspects, paying attention to the effects of internalized colorism and appearance-based discrimination on sexual agency and decision-making. This may look like adding colorism modules to existing sexual health programs implemented within schools, community organizations, and with families.

## 5. Conclusions

This rapid review highlighted that colorism operates as a meaningful social determinant of sexual health among Black and Brown women and girls. Across the identified studies, colorism appeared to affect sexual health through the interrelated pathways of internalization and self-esteem, colorist desirability hierarchies and sexual agency, and differential sexualization experiences based on skin tone. Although the evidence base is small, it consistently converged on these mechanisms across studies conducted in different contexts and populations and using varying methodological approaches. As such, the review highlights that the sexual health of Black and Brown girls and women cannot be fully understood or protected without research frameworks that acknowledge and account for the effects of colorism.

## Data Availability

No new datasets were generated or analyzed during the current study. All data supporting the findings of this review are available within the cited published literature.
